# Are Dual-Method Messages Undermining STI/HIV Prevention?

**DOI:** 10.1155/2011/691210

**Published:** 2011-11-15

**Authors:** Ann O'Leary

**Affiliations:** Division of HIV/AIDS Prevention, National Center for HIV, Hepatitis, Sexually Transmitted Diseases, and Tuberculosis Prevention, Centers for Disease Control and Prevention, 1600 Clifton Road, MS E-37, Atlanta, GA 30333, USA

## Abstract

Adolescent girls and young women who are at risk for unplanned pregnancy and sexually transmitted infection (STI), including HIV, are frequently counseled to use a hormonal contraceptive to protect against the former and condoms to protect against the latter, for exampe, American College of Obstetricians and Gynecologists, 2011. The present paper reviews the literature on multiple risk messages, compliance with this dual-use recommendation, predictors of dual use, and interventions developed to encourage dual use. Data indicate that simultaneous use of these two methods is not common, and that efforts to encourage dual use have not yielded promising results. An alternative is to recommend condom use alone, since condoms protect very well against STI and HIV, and quite well against pregnancy when used consistently and correctly. The availability of emergency contraception is relevant here. Research utilizing a randomized controlled trial is recommended.

## 1. Introduction: Why Recommend Dual Protection during Sex?

Unprotected sex can have numerous consequences, including unintended pregnancy and sexually transmitted infections (STI). Among US teens and young women, both are all too common and occur disproportionately within the same population: low-income women of color, especially African American women. In 2009, 410,000 teens aged 15–19 gave birth in the United States [1]. It is estimated that half of all pregnancies are unintended, and the figure is higher—62%—among women with incomes below the poverty level [2]. Poverty is also a risk factor for HIV/AIDS and other STIs [3, 4]. HIV incidence among those aged 13–29 is estimated to be 27% of the total 21,000 cases per year [5]. Data from the National Health and Nutrition Examination Survey (NHANES) indicated that, among sexually experienced girls aged 14–19, 38% had at least one of five STIs [6]. The rate for African American girls was 44%. It is critical that everything possible be done to reduce the prevalence both of unintended pregnancy and STI, the greatest risk for which is largely present among the same young women.

Hormonal contraceptives (HCs), which are highly effective at preventing pregnancy, do not prevent STI, including Human Immunodeficiency Virus (HIV). On the other hand, male condoms, if used correctly and consistently, greatly reduce transmission of HIV [7] and STI [8, 9]. In an effort to prevent both unintended pregnancy and STI, guidelines for adolescents recommend encouraging the use of both male condoms and another contraceptive: “… sexually active adolescents should be encouraged to use condoms in conjunction with a more effective method of contraception to provide both effective pregnancy prevention and protection against STIs” [10], although it is not known how compliant health care providers are with this recommendation. There are data to suggest that the use of some HCs may potentiate the risk of HIV infection for purely biological reasons, although study findings are inconsistent [11, 12]. On the other hand, condoms, which are highly effective in preventing sexually transmitted infection and transmission, are less effective than HCs in preventing pregnancy, both in typical and perfect use. Because many adolescents and young women are at risk for both unintended pregnancy and STI, it is frequently recommended that such girls and women be counseled to use two forms of protection, one to prevent pregnancy and one to prevent STI [10, 13, 14]. 

To our knowledge, no published research has tested directly how these two risk messages are processed or evaluated. A body of literature in social psychology, however, has studied the effects of multiple messages and found them to be complex [15]. Indeed, individual differences in the “need for cognition”—that is, tendency for and enjoyment of thought—can affect whether the second of two messages is even cognitively processed [16]. 

## 2. What Do We Know about Multiple Risk Messages?

When measures are used to protect people from harm, some recipients may increase the riskiness of their behavior in other ways. For example, there is evidence that children engage in more hazardous physical activity when wearing safety gear [17]. This phenomenon is known as “risk compensation”, a form of behavioral disinhibition that has been theorized to be a product of “risk homeostasis” [18]. In the area of HIV prevention, concern about risk compensation in the form of reduced condom use (“condom migration”) has been voiced in connection with preventive vaccines, preexposure prophylaxis, and vaginal microbicides [19, 20], all of which are assumed to provide only partial protection against HIV and all of which, at least currently, are recommended for use in conjunction with condoms. While to date evidence for risk compensation in these contexts has been mixed [21–26], concern remains high and the issue is widely believed to be unresolved [27]. 

Also relevant to this issue is the possibility that reasoning about dual-risk messages opens the door for post hoc justifications for risky behavior. For example, Gold and colleagues studied gay men who had engaged in risky sexual behavior [28, 29]. In these studies, men justified their risk behavior by reasoning such justifications as “AIDS is hard to transmit” and “I'm at less risk than most guys.” It may be that justifications are used to avoid condom use when another form of protection is in place and can be considered to confer safety (e.g., a microbicide; hormonal contraception).

Another form of multiple messages, “hierarchical messages” combines different HIV/STI risk reduction strategies to be used, not concurrently, but rather singly, in descending order of efficacy [30]. For example, if abstinence is not possible, use a male condom; if this is not possible, use a female condom; and so on. While this approach has difficulties, including the use of less effective strategies compared with single-method messages [31, 32], it is less relevant to the present topic and will not be extensively reviewed here.

## 3. How Often Are Dual-Methods Recommendations Followed?

A review of the literature on dual methods was conducted using the terms “dual method” and “dual protection” in Medline (1996—present) and PsycInfo (1987—present). Only studies conducted in the United States were used. Studies have yielded estimates of 3% to 26% of study samples that report dual method use, for periods of time ranging from last six to the previous six months [33–42]. It should be noted, however, that the study obtaining the rate of 26% included HIV-positive women and all participants were six months postpartum [23]. This study also distinguished between alternating and simultaneous dual-method use, the former describing use of different methods at different times and the latter indicating use of both methods on every sexual occasion. Of the women reporting dual use in this study, only 64% reported simultaneous use. This finding suggests that a sizeable proportion of women reporting dual use in the studies just cited are in fact engaging in alternating use, meaning that the rates of dual methods *as intended* are overestimated by studies not making this distinction.

Data on contraceptive and condom use are reported from the Youth Risk Behavior Survey, a nationally representative school-based health survey, by the Centers for Disease Control and Prevention [43]. In 2009, use of dual methods (a condom and an HC) at last intercourse was reported by 13.1% of white girls, 53.6% of black girls, and 3.2% of Hispanic girls. Condom use alone, however, was reported by 42.5%, 45.1%, and 44.9% of these groups, respectively. It should be noted that these respondents were aged 15–19, and that condom use decreases with age [44]. In a study of women aged 18–45, the pattern for dual use was reversed: 5% of white women, 15% of African American women, and 16% of Hispanic women were users of condoms in conjunction with an efficient method of birth control [39].

It should be noted that use of dual methods by women appears to be increasing, at least between 1995 and 2002 [45], although this effect is THE strongest for younger women (aged 15–17) and drops off at age 18-19 [45]. This increase, as well as a general increase in condom use and abstinence, are thought to account for declines in teen pregnancy rates [45].

### 3.1. Predictors of Dual Use

A number of factors have been shown to predict dual methods use. One factor is level of risk, and here the news is unfortunate: those at higher risk are less likely to use dual methods. In one study [33], women with six or more partners were less likely than those with fewer to use methods preventing STI (i.e., condoms). Those with good communication with parents [38, 46], whose sexual debut was later [46], and who had fewer nonsexual risk behaviors [46], have been found to be more likely to use dual methods. On the other hand, another study found that women with more than one sex partner were more likely to use dual methods than any single method [35]. Younger women are more likely to use dual methods than older women [35]. Thus, conflicting results have been obtained regarding this issue.

Aspects of sexual relationships also influence contraception and condom use. Those who report newer and less committed sexual relationships [35, 37, 38, 42] are more likely to be dual methods users than others. Similarly, married or cohabiting women are less likely to use dual methods (or condoms) [39, 42]. Conversely, women whose communication with sex partners was good and who expected good support from partners should they become pregnant [42] were also likely to be dual method users. Also perhaps counterintuitively, women who shared contraceptive decision-making with their partners were more likely than those who did not to use dual methods [39]. These correlational data are difficult to interpret; however, it may be that women who are relatively empowered in their relationships are able to use the methods that they choose. 

Not surprisingly, the most robust predictors of dual method use have to do with women's primary concerns; that is, avoiding pregnancy versus avoiding disease. In fact, in one study, relationship status was significant in bivariate analysis but lost significance when motives for use were entered into the model [46]. Those reporting high levels of motivation to avoid STI and HIV [32, 39] or perceive their partners to possibly have HIV or STI [38] are also more likely to be dual-method than HC-only users. In a study of adolescents, however, desire to avoid pregnancy was associated with dual method use, while concern about STI and HIV did not [34]. It should be noted that this comparison was with the combination of HC alone and condom use alone, and it cannot be determined which of these subgroups may have been responsible for the difference (i.e., HC and condom use may have “cancelled each other out” in the analysis, while condom use may, by itself, have been significantly associated with concern about STI and HIV). Finally, women who express concern about both pregnancy and STI, or have had an STI, or perceived that condoms were effective against STI, were more likely to use dual methods rather than a single method [39].

In the afore-mentioned study that included HIV-infected women, HIV positivity was a strong predictor of dual method usage [41], indicating that women were strongly motivated to prevent transmission of the virus to their partners. Women in this study, who were six months postpartum, were also more likely to report dual method use if they felt that having another pregnancy “would be upsetting” if it occurred within the next six months. Additionally, dual method use was significantly predicted by having abstained from alcohol during the postpartum period.

A study conducted in the Netherlands [36] used hypothetical vignettes to assess behavioral intentions to use condoms. In the vignettes, participants imagined that they had just met someone that they were mutually attracted to and with whom they had decided to have sex. In one vignette, it was stated that there would be no risk of pregnancy, the other contained no such statement. Not surprisingly, intention to have sex without a condom was three times as high for the vignette in which pregnancy was not a concern as for the other. In this study, each participant responded to both vignettes, and the investigators compared condom use intentions for those who changed from condom to noncondom responses when the one with pregnancy concern preceded the other. Those who abandoned condom use intentions differed from the intentions of those who did not in three ways: they had lower perceived seriousness of STI, lower perceptions of friends' perceived seriousness of STI, and they reported lower perceived susceptibility to STI after unprotected sex.

Thus, although studies have yielded some inconsistent results, it appears that most women use a single method of protection, and the method used is based on motivation to prevent the outcome of greatest concern [47]. This is most often concern about pregnancy, which is associated with HC use; those concerned about STI are more likely to be condom-only or dual-method users. 

## 4. Condoms to Prevent STI and Pregnancy: How Effective Are They?

It is very clear that condoms are effective against HIV [1] and STI [9], when used correctly and consistently. It is also the case that HCs have no appreciable effect on preventing STIs. But what about pregnancy? There is indeed evidence that condoms as typically used are a less effective means of contraception than HC [48]. Condoms, when used perfectly, have a pregnancy rate of 2% and when used typically, it is 17.4%. The pill, when used perfectly, has a pregnancy rate of 0.3% and when used typically, it is 8.7%. However, condoms are in fact superior to other commonly used alternative forms. As typically used, condom failure rates are on a par with those of diaphragms and the cervical cap or sponge for nonparous women and are markedly superior to withdrawal, spermicides, the female condom, and the cervical cap or sponge for parous women [48]. Using data from the National Survey of Family Growth, Pazol and colleagues [49] estimated that if half of all women using HCs only also used condoms, approximately 40% of unplanned pregnancies and abortions could be prevented. In any case, it is clear that many teens do use condoms, although existing data cannot tell us with what consistency. Condom use has been increasing in this age group, presumably in response to HIV/STI prevention programs and is believed to be responsible for decreases in teen pregnancy rates [1, 44]. To gauge the consistency with which condoms are used, it is important to assess use specifically with different partners [50]; however, consistent use appears to be suboptimal. For example, one study found that only 45% of condom-using teenage boys used condoms with every act of sex [44].

The use of male condoms appears to be a viable option for most women. On the other hand, achieving correct and consistent condom use on every occasion of sex is difficult. Interventions that convey knowledge and build skills regarding STI/HIV can be effective enough to prevent new STI infections for as long as a year [51–53]. Given the importance of motivations for women's choice of method described above, interventions that stress the prevalence and seriousness of HIV and other STI may be a useful adjunct to routine contraceptive counseling.

Concerns about unintended pregnancy, regardless of contraceptive method used, are further mitigated by the widespread availability, without prescription, of emergency contraception (EC) [54]. Laws and policies related to accessibility vary somewhat by state [55]. Emergency contraceptives work by preventing ovulation or fertilization, and possibly by preventing implantation of the fertilized egg, although this mechanism has not been supported by clinical data [55]. It has been reported that the contraceptive strategy of condom use with EC backup is increasing in prevalence since EC became available [56]. 

## 5. Interventions to Promote Dual Methods Use

A small number of behavioral interventions to increase use of dual methods have been tested. One trial [57], randomized black and Latina adolescent girls on HC either to a video, STI/HIV counseling based on Project RESPECT [52], both the video and counseling, or usual care. The video was designed to promote dual methods and increase perceived vulnerability to HIV. At a three-month follow-up, the women who had received both interventions were significantly more likely to report having used a condom during their most recent sexual encounter, compared with each of the other three groups. Unfortunately, this result was nonsignificant at the 12-month follow-up. This study suffered from severe attrition, with only 55% of participants returning for the three-month follow-up and 49% for the 12-month follow-up. This low-retention rate renders interpretation of the findings difficult.

An analysis of data from the Project RESPECT trial, a condom-focused multisite intervention study conducted in STI clinics, examined uptake of condoms by women in the trial who were using HC [58]. Among these women, condom use increased as a result of the intervention, and most women remained on their HC regimens. Thus, in response to a condom-focused intervention, many women became dual-methods users, although most did not use condoms consistently (i.e., were alternating dual method users).

A more recent study tested a computer-based intervention designed to encourage dual methods use among at-risk women [59]. Participants were at risk for either pregnancy or STI and were enrolled irrespective of type of contraception used. Women were randomized to receive this intervention or general contraceptive information. Based on self-report, the intervention increased the ever-use of dual methods. While the confidence interval of the unadjusted OR (1.38) contained 1.00, when adjusted for baseline differences using a propensity score, this effect became significant (although alternating versus simultaneous use was not assessed). Differences between treatment arms for consistent condom use, STI, unplanned pregnancy, and individual STIs were all nonsignificant. The authors interpreted these findings to suggest that the use of dual methods was not sustained long enough to prevent STI and pregnancy. Use of dual methods was predicted by higher education level, substance use, and use of either hormonal contraceptives or male condoms at baseline.

Finally, a recent study tested a provider-delivered intervention designed to promote dual method use by providing counseling both about STI risk and pregnancy risk, compared with a standard of care in which only pregnancy was addressed [60]. In both conditions, patients chose their preferred method without specific encouragement from the counselor. The primary outcome, rather than use of dual methods per se, was the number of sex acts unprotected by a male or female condom. The intervention group reported 3 fewer unprotected acts than the standard of care group, a difference that approached significance.

In summary, the research on interventions to promote dual-method use is mixed. Condom-focused interventions that focus attention on the threats of HIV and STI are successful in increasing condom use, even when women are on HC prior to the intervention. Other studies have yielded mixed results—that is, self-reported outcomes at odds with biological ones—and nonsustained or null results. These results suggest that dual methods counseling frequently fails to achieve its desired outcome, consistent and sustained use of HC and condoms at every act of intercourse. Since condoms protect very well against STI, and quite well against pregnancy, and given the availability of EC should condom failure occur, recommending consistent use of condoms may be more effective at preventing STI than recommending the use of dual methods. Moreover, if the method used must protect against the outcome of greatest concern for the woman, and if that outcome is pregnancy, she may be less tempted to abandon condom use if that is her only source of protection. On the other hand, if she is protected from pregnancy by hormonal contraceptives, she may be more tempted to forego condoms, particularly under male resistance. However, I believe that this hypothesis should be tested empirically, by comparing a dual-use recommendation with a condom-only one. I would hypothesize that women in the condom-only arm would acquire fewer STIs, but may need to use EC more often and may even become pregnant more often. In addition, characteristics of successful dual-method users could be identified, in a message-controlled context.

Would such a trial be feasible? Would young women be willing to be randomized to the form of protection that they would use for a prolonged period? Recent evidence suggests that the answer to this question is “yes.” A feasibility study to assess the acceptability of an RCT to examine whether the use of hormonal contraceptives creates biological vulnerability to STIs has been conducted [61]. In this study, potential at-risk participants were asked whether they would be willing to be randomized to hormonal contraceptives or to an IUD. They were also asked to provide urine or endocervical swabs for STI testing. Overall, about 70% of participants said that they would be willing to participate in this trial, indicating that women are willing to be randomized, and suggesting that such a trial is indeed feasible.

Would such a trial be ethical? Of course, informed consent would be obtained, and participants will understand that they can cease participation in the trial (or switch arms?) without consequence. To ensure that participants (and parents/guardians?) were fully informed and willing to be randomized to either intervention, investigators would explain to participants the advantages and disadvantages of condoms and HC as tools for pregnancy and STI prevention. 

The ethical sticking point appears to be, for some, randomizing participants to receive a less effective method of contraception. However, the *overall* risks and benefits to study participants must be weighed to determine whether equipoise exists between study arms. On the one hand, the literature indicates that the condoms-only group would receive a less effective pregnancy prevention message than the dual-method (standard of care) arm. Therefore the apparent risk of unintended pregnancy would be increased in the condoms-only arm. But on the other hand, the condoms-only message may be more effective than the dual methods message for preventing STIs, including HIV. (Could be noted here or elsewhere the negative health consequences of STI acquisition among teenagers.) The potential benefit of STI avoidance could balance or outweigh the increased risk of failed contraception. Moreover, participants would be counseled on emergency contraceptive use and given access to this method in cases of failed contraception. This counseling and access would reduce the risk of unintended pregnancy among all study participants and mitigate the increased risk in the condoms-only arm. Although the recommendation to adolescents and young women at risk for unplanned pregnancy and STI is to use both a hormonal contraceptive to prevent pregnancy and male condoms to prevent STI, there is evidence to suggest that this approach is failing, and if this is so, that a significant, unnecessary burden of STI, including HIV, may be the result. This hypothesis should be tested empirically, using a rigorous design. 

## 6. Looking to the Future: Dual Protection Technologies

It is possible that this issue will be solved by the development of technologies that prevent both pregnancy and STI. The global fight against HIV has led to great efforts to develop topical microbicides that can be applied vaginally to neutralize the virus before it can infect the woman. The premise for the importance of such products was that, since men control condom use and often may not agree to their use, women needed methods to prevent STI/HIV infection that were under their own control and undetectable by partners. The first such candidate was in fact a spermicidal surfactant, nonoxynol-9, which was shown to kill HIV and other sexually transmitted pathogens *in vitro*. Unfortunately, this product caused *in vivo* damage to the epithelium, permitting the entrance of HIV, and actually *increased* the likelihood of infection with the virus [62]. A more recent attempt to create a dual-method product, an acid-buffering gel, showed promise as dual-protection agent [63] but unfortunately was shown to be ineffective against HIV.

Efforts to develop an effective microbicide have continued, and currently focus on antiretroviral products [64]. Recently, the CAPRISA trial of a vaginal gel containing the antiviral tenofovir was shown to reduce HIV incidence by between 38% and 54%, depending on adherence, compared to a placebo [65]. This product does not prevent pregnancy; in fact a pregnancy rate of 4.0 per 100 women-year was observed. Currently, most products in trials are ones that are hoped to enable pregnancy, and are thus not dual-protection agents. However, if an effective microbicide is identified, efforts to create one that also prevents pregnancy will be the next step [66]. 

## 7. Conclusion

Until fully effective dual-protection technologies become available, we will continue to face the conundrum of the dual-method message. Should the recommended trial yield the hypothesized results, an argument could be made for recommending condom-only contraception, and the study results could be informative as to the characteristics of successful dual-method users.
